# Prevalence of potential drug–drug interactions in outpatients of a general hospital in China: a retrospective investigation

**DOI:** 10.1007/s11096-020-01068-3

**Published:** 2020-06-03

**Authors:** Weifang Ren, Yujuan Liu, Jun Zhang, Zhonghong Fang, Huan Fang, Yuan Gong, Xiaoqun Lv

**Affiliations:** Department of Pharmacy, Jinshan Hospital, Fudan University, Shanghai, China

**Keywords:** Outpatient, Polypharmacy, Drug–drug interactions, Prescription, China, Potential interactions

## Abstract

*Background* Potential drug–drug interactions are important factors resulting in adverse drug reactions or therapeutic failure. Therefore, potential drug–drug interactions need to be identified to prevent the related risk and improve drug safety. *Objective* This study was designed to determine the prevalence of potential drug–drug interactions and investigate the association of potential drug–drug interactions with characteristics in outpatient prescriptions. *Setting* A large-scale general university hospital in Jinshan District of Shanghai, China. *Method* The retrospective study was conducted on data obtained from prescriptions containing two or more drugs, written for outpatients older than 18 years. They were screened for potential drug–drug interactions using Lexi-Interact in UpToDate, Stockley’s Drug Interactions and Medicine Specification in the order of priority. *Main outcome measure* Drug–drug interactions with C, D, X risk rating and clinical parameters recorded at the prescriptions. *Results* 16,120 prescriptions were screened for the presence of potential drug–drug interactions and 4882 (30.29%) prescriptions containing 6667 potential drug–drug interactions were identified. Among 6667 potential drug–drug interactions, 90.81% (6054/6667), 8.49% (566/6667), 0.70% (47/6667) potential drug–drug interactions belonged to the risk category of C, D and X, respectively. Male, old age and polypharmacy increased the likelihood of potential drug–drug interactions. The most frequently prescribed drugs responsible for potential drug–drug interactions included pioglitazone, dihydrocodeine, thalidomide, sotalol, amiodarone and amlodipine. The predominant potential adverse outcome of potential drug–drug interactions was the increased central nervous system suppression function with the mechanism of reinforced pharmacological effects. *Conclusion* This study showed that potentially significant drug–drug interactions in outpatients were prevalent in real-world practice. Considering the risk of potential clinical consequences related to potential drug–drug interactions, it is necessary to implement the computerized surveillance and warning systems with drug–drug interactions databases as well as develop the clinical guidelines regarding the widespread potential drug–drug interactions.

**Electronic supplementary material:**

The online version of this article (10.1007/s11096-020-01068-3) contains supplementary material, which is available to authorized users.

## Impacts on practice

- Drug–drug interactions are frequent in outpatients. Health professionals including physicians and pharmacists should raise awareness of the potential impact of drug–drug interactions.
- It is important to incorporate the clinical pharmacists into the healthcare team to routinely screen the potential drug–drug interactions.
- A computerized warning systems with smarter screening software may be beneficial in reducing the potential risk of drug–drug interactions.
- Further research is needed to develop clinical guidelines regarding the widespread potential drug–drug interactions along with their potential adverse outcomes and management strategies.

## Introduction

Potential drug–drug interactions (pDDIs) are defined as reactions likely to occur that may alter the effect or safety of two or more drugs concomitantly administered [1]. Drug–drug interactions (DDIs), which induce approximately 22% of drug withdrawal and adverse drug reactions-related hospital admissions, are one of the preventable drug related problems with the risk of deteriorating the therapeutic effect, causing adverse drug reactions and resulting in treatment failure or even death [2]. Therefore, it is of great significance to identify pDDIs to prevent the related risk and improve the clinical medication safety.

Several factors contribute to the occurrence of DDIs in populations, such as age, comorbidities, polypharmacy, nutritional status, and genetic constitution of an individual [3, 4]. Therefore, DDIs are of concern especially in elderly patients with comorbidities. It has been reported that the risk of adverse drug reactions resulting from DDIs increased 50% in people taking five medications, and 100% in those taking eight drugs [5].

DDIs are highly prevalent not only in outpatients but also in hospitalized patients especially in ICU [6, 7], oncology [8–10] and hematology [11, 12]. The prevalence of pDDIs varies from 16% to 96% in different studies [13–16]. Given the lack of investigation on the epidemiology of DDIs in the outpatients of Jinshan Hospital, Fudan University, the present study aimed to gain insight into the real-world prevalence of pDDIs in outpatient prescriptions and classify the severity of the interactions.

## Aim of the study

The aim of this study was to determine the prevalence of pDDIs in outpatients by screening the prescriptions using Lexi-Interact in UpToDate, Stockley’s Drug Interactions and Medicine Specification in the order of priority. Besides, we investigated the association of pDDIs with variables in prescriptions.

## Ethics approval

Ethical approval was obtained from the Ethical Committee of Jinshan Hospital, Fudan University (IEC-2020-S05). As the study was conducted from prescription records, individual informed consent was not applicable.

## Method

This cross-sectional retrospective study was conducted in the outpatients of Jinshan Hospital, Fudan University, which is a large-scale general hospital integrating medical service, education and research. The prescriptions for analysis were collected from June 1st to July 1st in 2019. The study subjects were all outpatients older than 18 years, treated with at least two drugs. The data included diagnosis, demographic data (such as age and gender), prescribed drugs with the exclusion of herbal medicines due to rare knowledge of drug–herbal interactions and the high compositional variability inherent to herbal drugs [17]. Compound preparations which contain multiple pharmacologically active ingredients were analyzed individually according to each ingredient.

pDDIs have been examined based on the Lexi-Interact in UpToDate and classified into five categories according to the clinical significance as A (no known interaction), B (no action needed), C (monitor therapy), D (consider therapy modification) and X (avoid combination). If the drugs were not included in Lexi-Interact, we referred to Stockley’s Drug Interactions and Medicine Specification. The priority of retrieving order was Lexi-Interact, Stockley’s Drug Interactions and Medicine Specification.

The data were recorded in a Microsoft Office Excel file. Descriptive statistics were applied to analyze the demographics of total outpatients, number of drugs, and pDDIs characteristics. The values were presented as numbers and percentages as appropriate. To investigate the potential risk factors of pDDIs, the logistic regression was performed to calculate odds ratios (ORs) and their 95% confidence intervals (95% CIs) of pDDIs based on the patient’s characteristics. Binary variables were defined for prescriptions exposing to at least one C-, D-, or X-interaction. The effect of gender on the occurrence of pDDIs was analyzed in the study. Categories for age were defined as follows: young (18–39), middle-aged (40–64), and elderly (≥ 65). Categories for number of drugs in each prescription were defined as 2–3, 4–6, and 7 or more. Another variable in the analysis was patient sex. A *p* value of < 0.05 was considered as statistically significant. All data analyses were performed using the SPSS statistical software (version 18.0, IBM, USA).

## Results

### Population characteristics

A total of 16,120 prescriptions were evaluated for the presence of pDDIs. Clinical data of the study population was provided in Table 1. Male patients comprised 48.68% of the study population. Mean age of the patients was 57.30 ± 16.11 years, where 36.11% of the patient population were elderly individuals aged ≥ 65 years as well as young patients of age < 40 years and middle-aged patients (40–64) accounted for 16.67% and 47.22% respectively. The most commonly number of medications prescribed for each patient was no more than six, accounting for 94.89%.

**Table 1:** Characteristics of the patients with potential drug–drug interactions and factors associated with the presence of potential drug-drug interactions

Characteristics	No. of patients (%)	No. of patients with pDDIs (%)	%^a^	Adjusted OR (95% CI)	*p* value
Sex					
Male	7847(48.68)	2526(51.74)	32.20	Reference	
Female	8273(51.32)	2356(48.26)	28.47	0.85(0.79-0.91)	< 0.01
Age(years)					
Young (18–39)	2687(16.67)	509(10.43)	18.94	Reference	
Middle-aged(40–64)	7612(47.22)	2389(48.93)	31.38	2.03(1.82–2.26)	< 0.01
Elderly (≥ 65)	5821(36.11)	1984(40.64)	34.08	2.22(1.98–2.49)	< 0.01
Numbers of prescribed medicines					
≤ 3	10,264(63.67)	2463(50.45)	23.98	Reference	
4–6	5032(31.22)	2114(43.30)	42.07	2.26(2.10–2.43)	< 0.01
≥ 7	824(5.11)	305(6.25)	36.89	2.10(1.81–2.45)	< 0.01

^a^Percent value means a percentage of patients with potential drug–drug interactions in total number of patients within the group

### Prevalence of pDDIs and factors associated with pDDIs

**Fig. 1:**
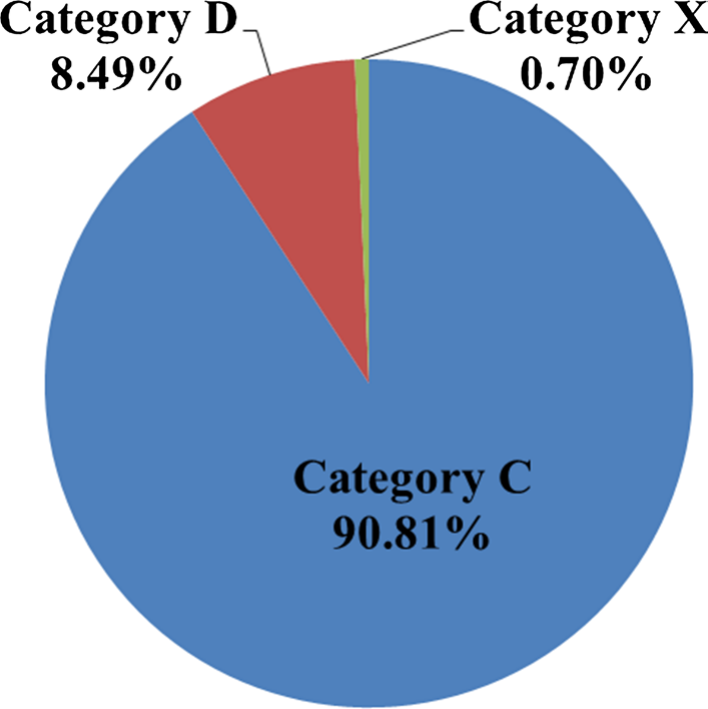
Distribution of the identified potential drug–drug interactions (*n *= 6667), including the risk category C, D and X

A total of 4882 prescriptions (30.29%) were identified with 6667 pDDIs. And pDDIs fell into category C, D, and X in a proportion of 90.81% (6054/6667), 8.49% (566/6667) and 0.70% (47/6667), respectively (Fig. 1).

The logistic regression showed that sex, age and number of prescribed medicines were independently associated with the occurrence of pDDIs (p < 0.01) (Table 1). Female patients had lower risk of developing pDDIs compared with the male patients (OR 0.85, 95% CI 0.79–0.91). Middle-aged and elderly patients were found to have the risk of pDDIs increased by 2.03 (95% CI 1.82–2.26) and 2.22 (95% CI 1.98–2.49), respectively. The number of prescribed medicines was also a risk factor for the occurrence of pDDIs (*p *< 0.01).

Statistical analysis showed that 1201 prescriptions (24.6%) were identified with more than one pDDIs even up to 6 pDDIs per prescription, 761 (15.59%) prescriptions with 2 pDDIs per prescription and 341 (6.98%) prescriptions with 3 pDDIs per prescription (Table 2). The characteristics and distributions of those prescriptions were shown in Table 2.

**Table 2:** Distribution of the characteristics among the prescriptions with different number of potential drug–drug interactions per prescription

Characteristics	No. of pDDIs per prescription, *n* (%)					
	1	2	3	4	5	6
Sex						
Male	1840	435	200	32	18	1
Female	1841	326	141	28	15	5
Age(years)						
Young (18–39)	437	48	23	1	0	0
Middle-aged (40–64)	1830	376	141	24	16	2
Elderly (≥ 65)	1414	337	177	35	17	4
Number of prescribed medicines						
≤ 3	2055	308	100	0	0	0
4–6	1360	424	234	58	32	6
≥ 7	266	29	7	2	1	0
Total	3681	761	341	60	33	6
	(75.40)	(15.59)	(6.98)	(1.23)	(0.68)	(0.12)

### Potential clinical consequences and mechanisms of pDDIs

Table 3 demonstrated the potential clinical consequences and related mechanisms of top 20 pDDIs in category D. The most frequent drug pairs of pDDIs with category D were pioglitazone-glimepiride (34 cases). The most frequent potential clinical consequence of the identified pDDIs was enhanced central nervous system suppression function. The predominant mechanism was reinforced pharmacological effects (57.64%).

**Table 3:** Common (top 20) potential drug–drug interactions belonging to Category D with the potential clinical consequences and related mechanism

Interacting pair	Patients: n (%)	Potential clinical consequences	Mechanism of interaction
Pioglitazone–glimepiride	34(6.01)	Hypoglycemia	Reinforced pharmacological effects (pharmacodynamics)
Pioglitazone–insulin	24(4.24)	Hypoglycemia, increased fluid retention and heart failure effect	Reinforced pharmacological effects (pharmacodynamics)
Propafenone–sotalol	23(4.06)	QT-interval prolongation	Metabolic inhibition (pharmacokinetic)
Baclofen–dihydrocodeine	23(4.06)	Enhanced central nervous system suppression function	Reinforced pharmacological effects (pharmacodynamics)
Amiodarone–warfarin	22(3.89)	Potentiated warfarin plasma concentrations and anticoagulant effect	Metabolic inhibition (pharmacokinetic)
Eperisone–dihydrocodeine	20(3.53)	Enhanced central nervous system suppression function	Reinforced pharmacological effects (pharmacodynamics)
Flunarizine-codeine	19(3.36)	Enhanced central nervous system suppression function	Reinforced pharmacological effects (pharmacodynamics)
Fluoxetine–perphenazine	19(3.36)	Increased plasma concentrations of perphenazine	Metabolic inhibition (pharmacokinetic)
Tizanidine–dihydrocodeine	18(3.18)	Enhanced central nervous system suppression function	Reinforced pharmacological effects (pharmacodynamics)
Chlorpheniramine–dihydrocodeine	17(3.00)	Enhanced central nervous system suppression function	Reinforced pharmacological effects (pharmacodynamics)
Simvastatin–amlodipine	16(2.83)	Increased plasma concentrations of simvastatin, risk of myopathy and rhabdomyolysis	Metabolic inhibition (pharmacokinetic)
Calcium acetate–alendronate sodium	15(2.65)	Decreased absorption and plasma concentrations of alendronate sodium.	Formed insoluble compounds (pharmacokinetic)
Valproate–lamotrigine	14(2.47)	Enhanced lamotrigine plasma concentrations.	Metabolic inhibition (pharmacokinetic)
Tizanidine–codeine	12(2.12)	Enhanced central nervous system suppression function	Reinforced pharmacological effects (pharmacodynamics)
Testosterone undecanoate-ciclosporin	12(2.12)	Increased hepatotoxicity and plasma concentrations of testosterone undecanoate	Unknown
Fluoxetine–clopidogrel	11(1.94)	Decreased plasma concentrations of clopidogrel active metabolite	Metabolic inhibition (pharmacokinetic)

As shown in Table 4, the majority of pDDIs with category X were related to thalidomide with the potential clinical consequence of enhanced central nervous system suppression function through the mechanism of reinforced pharmacological effects. The most frequent interaction found in category X was the simultaneous administration of ebastine and thalidomide.

**Table 4:** Common (top 10) potential drug–drug interactions belonging to Category X with the potential clinical consequences and related mechanism

Interacting pair	Patients: *n* (%)	Potential clinical consequences	Mechanism of interaction
Ebastine–thalidomide	7(14.89)	Enhanced central nervous system suppression function	Reinforced pharmacological effects (pharmacodynamics)
Levocetirizine–thalidomide	5(10.64)	Enhanced central nervous system suppression function	Reinforced pharmacological effects (pharmacodynamics)
Doxazosin–tamsulosin	4(8.51)	Antihypertensive effects	Reinforced pharmacological effects (pharmacodynamics)
Sotalol–flupenthixol	4(8.51)	QT-interval prolongation	Reinforced pharmacological effects (pharmacodynamics)
Ketotifen–thalidomide	4(8.51)	Enhanced central nervous system suppression function	Reinforced pharmacological effects (pharmacodynamics)
Mizolastine–thalidomide	3(6.38)	Enhanced central nervous system suppression function	Reinforced pharmacological effects (pharmacodynamics)
Cetirizine–thalidomide	3(6.38)	Enhanced central nervous system suppression function	Reinforced pharmacological effects (pharmacodynamics)
Urokinase–heparin	3(6.38)	Potentiated anticoagulant effect	Reinforced pharmacological effects (pharmacodynamics)
Amiodarone–flupentixol	2(4.26)	QT-interval prolongation	Metabolic inhibition (pharmacokinetic)
Ibuprofen–celecoxib	2(4.26)	Increased toxicity	Reinforced pharmacological effects (pharmacodynamics)

## Discussion

This retrospective real-world study showed that among the 16,120 prescriptions screened by Lexi-Interact, 30.29% had at least one potentially DDI in the outpatient department of Jinshan Hospital, Fudan University with 90.81%, 8.49%, 0.70% pDDIs falling into the risk category of C, D and X, respectively. We identified that male, old age and polypharmacy increased the risk of pDDIs. The most frequent potential clinical consequence of category D and X pDDIs was enhanced central nervous system suppression function with the mechanism of reinforced pharmacological effects.

A cross-sectional study conducted by Mohammad Ismail et al. had showed that 22.3% of outpatients had pDDIs in a tertiary care hospital of Peshawar, Pakistan [14]. Janja Jazbar et al. conducted a retrospective nationwide study in Slovenia and reported 24.1% of the population was exposed to pDDIs with 9.3% D or X risk rating [18]. An institution-based cross-sectional study in Northwestern Ethiopia found that 34.5% of prescriptions with two or more medicines had at least one pDDIs [19]. The discrepancy among the prevalence of pDDIs in these studies may be attributable to the study design, drug prescribing pattern, screening system, criteria of pDDIs, and so on.

In our study, a higher percentage of pDDIs was found in male, which was in line with the previous findings [19, 20]. However, there are inconsistent results regarding the impact of gender on pDDIs. A cross-sectional study conducted in the Brazil found that being male was associated with a lower likelihood of pDDIs [21]. Besides, other studies found no significant difference in terms of sex [3, 22]. The cause of inconsistent results may be due to the study design and the longer lifespan in women [23].

In the present study, age was another variable associated with higher occurrence of pDDIs, specifically elderly aged over 65 compared to the young (18–39), which was in accordance with the previous study [19]. Due to concomitant ailments, the elderly patients take higher number of drugs at a time. Furthermore, they are more sensitive to pharmacokinetic effects.

As expected, the result of logistic regression analysis showed that polypharmacy (more than 3 medications per prescription) was a risk factor for the occurrence of pDDIs, which was in agreement with the pilot study of hospitalized pediatric patients conducted by Fabiola Medina-Barajas et al. [4]. Several studies had drawn a consistent conclusion that the increasing amount of medications was a risk factor for presenting drug interactions [24–26].

The results of the present study showed that a dominant proportion of identified pDDIs were found to be involved with the drugs of cardiovascular system, nervous system and endocrine system such as pioglitazone, propafenone, valproate, sotalol, amiodarone, warfarin, fluoxetine, clopidogrel, thalidomide, amlodipine, dihydrocodeine and codeine. Some previous studies showed inconsistent consequences. It was identified that levofloxacin, diclofenac, aspirin and clopidogrel were the dominant drugs for DDIs in outpatients [6], while Netsanet Diksis et al. had reported that aspirin, clopidogrel and enalapril were the three main drugs that caused pDDIs among hospitalized cardiac patients [27]. The reason for these contradictions may be due to variable drugs utilization pattern and the resources of drug information.

The most common interaction of category D and X in our study was pioglitazone + glimepiride, ebastine + thalidomide, respectively. Enhanced central nervous system suppression function was the most frequent clinical consequences of the identified pDDIs. However, the previous study found that the most common interactions in outpatient department were ibuprofen + levofloxacin, ciprofloxacin + diclofenac, aspirin + atenolol, and diclofenac + levofloxacin with the potential adverse outcomes such as seizures, bleeding, QT-interval prolongation, arrhythmias, tendon rupture, hypoglycemia/hyperglycemia and so on [14]. This inconsistency may be attributed to DDIs screening system and different drugs prescribing pattern.

This is the first study to analyze the prevalence of pDDIs in the outpatients of Jinshan Hospital, Fudan University, which nearly covers all clinical departments. However, there were some limitations in our study. First, this was a retrospective study which failed to evaluate the real-life clinical consequences of pDDIs. Besides, as a single-center study, the treatment regimens and the patient profile may not be generalizable. Second, the information of each patient was restricted to only one prescription sheet. We did not obtain totality data such as herbal medicines, OTC drugs and vitamins taken by outpatients, which might contribute to the underestimation of pDDIs. Third, there are several available DDI databases with the disparities in the inclusion of pDDIs and the severity grading [28]. However, only one screening database was employed to estimate the prevalence of pDDIs. To improve the precision of the detection, it is suggested that two or more screening databases should be used to evaluate pDDIs.

Clinical guidelines are generally applicable to single disease. However, the cumulative impact from multiple clinical guidelines is rarely considered [29]. Hence, it is necessary to develop clinical guidelines regarding the widespread pDDIs along with their potential adverse outcomes and management strategies [14]. Furthermore, this study will raise awareness of the importance of implementing the computerized warning systems with smart DDI databases as well as incorporating the clinical pharmacists into the healthcare team to routinely screen the pDDIs.

## Conclusion

This study demonstrated the real-world prevalence of pDDIs in outpatients at a rate of 30.29% with the most common interaction of category C. Gender, age and polypharmacy were risk factors significantly related to the occurrence of pDDIs. Further, enhanced central nervous system suppression function was most frequently observed as potential clinical consequences of the pDDIs in our study. Therefore, it is necessary to implement appropriate strategies so as to avoid or reduce these relevant pDDIs, such as computer-based warning systems of pDDIs, serum concentration monitoring, regular follow-up and accelerated clinical guides.

## Electronic supplementary material

Below is the link to the electronic supplementary material.

Supplementary material 1 (XLS 1494 kb)

## Data Availability

The datasets generated during and/or analysed during the current study are available from the corresponding author on reasonable request.
